# Estrogen Protects against Renal Ischemia-Reperfusion Injury by Regulating Th17/Treg Cell Immune Balance

**DOI:** 10.1155/2022/7812099

**Published:** 2022-10-06

**Authors:** Yang Zhang, Yuechen Chang, Ziwei Han, Ketao Ma, Xiansi Zeng, Li Li

**Affiliations:** ^1^Research Center of Neuroscience, Jiaxing University Medical College, Jiaxing, 314001 Zhejiang, China; ^2^Department of Physiology, Shihezi University Medical College, Shihezi, 832002 Xinjiang, China; ^3^Department of Anesthesiology, Affiliated Hospital of Jining Medical University, Guhuai Road, Jining, 272000 Shandong, China

## Abstract

Inflammation is a critical mediator of renal ischemia-reperfusion (I/R) injury (IRI), and T lymphocytes exert a key role in the renal IRI-induced inflammation. Connexin 43 (Cx43) is related to the maintenance of T lymphocyte homeostasis. Various preclinical researches have reported that estrogen is a renoprotective agent based on its anti-inflammatory potential. The present research is aimed at studying the role of T lymphocytes activated by Cx43 in 17*β*-estradiol-mediated protection against renal IRI. Female rats were classified into six groups: control rats, I/R rats, ovariectomized rats, ovariectomized I/R rats, and ovariectomized rats treated with 17*β*-estradiol or gap27. Levels of serum creatinine (Scr) and blood urea nitrogen (BUN) and Paller scoring were dramatically increased in I/R rats, especially in ovariectomized rats. By contrast, these indicators were markedly decreased by administering estradiol or gap27. Immunofluorescence staining revealed that CD4^+^ T cells infiltrated kidney tissues in the early stage of IRI. In both peripheral blood and renal tissue, the proportion of CD3^+^CD4^+^ T cells and ratio of CD4^+^ to CD8^+^ were high in I/R rats, especially in ovariectomized rats. The proportion of CD3^+^CD8^+^ T cells was low in peripheral blood but high in renal tissues. Administration of estrogen or Gap27 reversed these effects. IL-17 levels in both serum and tissue homogenate were significantly increased in ovariectomized rats subjected to I/R but significantly decreased in estrogen or gap 27 treated rats. The opposite trend was observed for IL-10 levels. Correlation analysis demonstrated that IL-17 was correlated positively with BUN, Scr, and Paller scores, while IL-10 was negatively correlated with these indicators. Western blot showed that Cx43 expression was markedly increased in the peripheral blood T lymphocytes of I/R rats, especially ovariectomized rats. After intervention with estrogen and gap27, Cx43 expression was significantly downregulated. These findings indicate that Cx43 may participate in the regulation of Th17/Treg balance by estrogen against renal IRI.

## 1. Introduction

Renal ischemia-reperfusion (I/R) injury (IRI) occurs clinically in different circumstances, such as partial nephrectomy [1], renal transplantation [2], complex cardiovascular surgeries requiring cardiac arrest [3, 4], postresuscitation shock [5], and chemical agent/drug-induced renal toxicity [6]. It is the most familiar cause of the development of acute renal injury and an important influential factor for kidney transplant prognosis [2, 7]. Although the pathophysiological process of renal IRI is complex, accumulating evidence suggests a critical role of the inflammatory response [8, 9].

Renal IRI is an inflammatory disease mediated by both the adaptive and innate immune systems [9, 10]. As an acute tissue injury, renal I/R injury leads to infiltration of immune cells, which contributes to both the damage and repair processes together with resident immune cells [2, 9, 11]. Numerous studies have shown that T cells exploit extremely complex functions in renal ischemic response [10, 12, 13]. A few studies utilizing selective knockout rodents and CD4^+^ and/or CD8^+^ reconstituted T cell subsets have implicated that CD4^+^ cells promote damage during the early stage after ischemia [12, 14]. CD4^+^ T cells could differentiate into T helper 1 (Th1), Th2, Th17, and regulatory T cell (Treg) subsets according to their functions under the stimulation of antigens and inflammatory factors. Different CD4^+^ T lymphocyte subpopulations related to promoting IRI and repair have been identified [15]. Most recently, several lines of evidence suggest that disruption of Th17/Treg cell immune homeostasis is closely associated with the development of many autoimmune and inflammatory diseases [16, 17]. In renal ischemic rats exposed to a high-salt diet, activated T cells, including IL-17-producing Th17 cells, were increased and potentially contributed to inducing salt-sensitive hypertension [18]. Treg is a subset of T helper cells and has potent suppressive effects [19, 20]. Those researches indicate that therapeutic strategies to decrease Th17 and other injurious T cell subtypes or to enhance Tregs would be profitable in promoting a healthy outcome following renal IRI. However, the mechanism deserves further investigation.

Gap junctions (GJs), intercellular channels at the cytomembrane, support the adjacent cells to communicate directly. Each of the two contacting cells provides one hexameric hemichannel (HC), namely, connexon, to form GJ channel [21, 22]. Connexin- (Cx-) based gap junction channel (GJC) or HC participates in maintaining the homeostasis of the immune system [21, 23]. Alterations in the expression of various Cxs or disruption of Cx-mediated cellular communication between T lymphocytes contributes to tissue remodeling or inflammation [24, 25]. Cx43 is a component of GJs. GJs formed by connexin 43 (Cx43) play significant roles in the T lymphocyte-triggered inflammatory response [26–28]. Furthermore, a recent study suggests that Cx43 is also involved in immunoglobulin secretion and cytokine production [23, 29]. However, the action of Cx43 in T lymphocytes in the renal IRI-driven inflammatory response is indistinct.

According to numerous epidemiological studies, there is significant gender difference in renal IRI with much lower incidence in female [30]. Interestingly, the incidence of renal IRI in females prior to menopause is lower than that in postmenopausal females. Those discrepancies can be explicated by the different levels in female sex hormone (estrogen) [31]. Clinical and experimental researches have demonstrated a role of estrogen in the protection of the kidney against ischemic renal disease [30, 31]. Estrogen signaling in CD4^+^ T cells inhibits Th17- or Th1-mediated inflammation in mice with colitis [32]. Physiological levels of estradiol (E2), the major estrogen secreted by the ovary, have been demonstrated to drive the maturation of immunosuppressive Treg differentiated from naive CD4^+^CD25^+^ T lymphocytes in murine [33]. Taken together, these findings indicate that estrogen exerts important roles in maintaining the homeostasis of immune cells. However, little is known about how estrogen influences T cell differentiation and inhibits the development of inflammation induced by renal IRI. Therefore, in this study, we investigated whether estrogen protects the kidney against I/R-mediated inflammation by regulating Cx43-mediated gap junctional intracellular communication (GJIC) in the T lymphocytes in peripheral blood. Confirming the cellular mechanisms underlying the modulating function of estrogen in renal IRI-mediated inflammation could provide a breakthrough for clinical therapy of IRI in future.

## 2. Materials and Methods

### 2.1. Drugs and Reagents

17*β*-Estradiol (E8875-1G) and cottonseed oil (C7767-1L) were obtained from Sigma-Aldrich (St. Louis, MO, USA). The specific gap junction blocker Gap27 (A1045) was procured from APExBIO (Houston, TX, USA). Due to the fat-soluble nature of 17*β*-estradiol, its powder cannot be directly miscible with cottonseed oil, so 10 mg of 17*β*-estradiol was first dissolved in 5 mL of absolute ethanol, and then, 45 mL of cottonseed oil was added to prepare 200 *μ*g/mL of 17*β*-estradiol. The mother liquor was stored in a 4°C refrigerator protected from light for later use.

### 2.2. Animal Preparation

Female adult Sprague-Dawley rats, aged 8-10 weeks and weighing 220-260 g and obtained from the Xinjiang Medical University Center for Animal Experiment (Urumqi, China), were employed in this study (License No. SCXK New 2003-0001). Rats were exposed to a 12 light/dark cycle and had free access to water and standard rat feed. Rats (8-10 weeks old) were subjected to ovariectomy. The rat ovariectomized model was established as described previously [30]. Briefly, rat ovaries were ligated and excised by bilateral paravertebral incisions after overnight fasting before surgery. After two weeks, renal IRI was achieved by removing the right kidney and clamping the left kidney pedicle for 45 min, followed by reperfusion for 6 hours [34].

### 2.3. Experimental Groups

Rats were divided randomly into six groups: the control rats, I/R rats, ovariectomized rats (OVX group), ovariectomized I/R rats (OVX + I/R group), and ovariectomized rats treated with 17*β*-estradiol (100 *μ*g/(kg·day), subcutaneously; OE + I/R group) or gap27 (25 *μ*g/(kg·day), intraperitoneally; OG + I/R group) 3 days prior to I/R surgery. All experimental animals were fasted for 12 hours before surgery and had free access to water. Each group had 8 rats, with a total of 48 rats.

### 2.4. Renal Functional Parameters

Blood samples were collected for assessment of renal function at 6 hours after reperfusion. Levels of BUN and creatinine in serum were assayed by using an automatic biochemical analyzer Modular DPP-H7600 (Roche, Shanghai, China).

### 2.5. Histopathological Measurement

After reperfusion for 6 hours, the animals were immediately anesthetized and subjected to 4% neutral buffered formalin (NBF; G2161, Solarbio, Beijing, China) perfusion by cardiac injection. Rat kidneys from the different groups were isolated immediately and were preserved in 4% NBF. Subsequently, hematoxylin and eosin (H&E) staining was performed to observe morphological changes of rat kidney sections. Quantification of renal slides was performed using the Paller scoring method [34].

### 2.6. Flow Cytometry Analysis

Surface antibody staining for peripheral blood T lymphocytes was performed as previously reported [35]. Briefly, after isolated from rat whole blood using erythrocyte lysis buffer (lysing solution 10x concentrate; 349202, BD, USA), peripheral blood mononuclear cell (PBMC) samples were double-stained with an APC-anti-CD4 (17-0048-41, eBioscience, CA, USA) or PE-anti-CD8a (12-0081-83, eBioscience, CA, USA) marker in combination with FITC-anti-CD3 mAb (11-0032-82, eBioscience, CA, USA) and analyzed by flow cytometry. Similarly, PBMC samples were incubated with all anti-rat APC-CD4, PE-CD25 (22-8425-71, eBioscience, CA, USA), and FITC-CD3 mAb.

The kidneys were collected and digested according to an established method as previously described [36]. Kidney-infiltrating lymphocytes were isolated and stained for flow cytometry analysis according to the manufacturer's instructions. For each experiment to detect cell surface antigens using multicolor fluorescent antibodies, unstained and single-color compensation controls were employed to eliminate any possible spillover of individual fluorescence.

Cell isolation and staining were conducted as described above [23, 37]. For intracellular cytokine staining of IL-17A (11-7177-81, eBioscience, CA, USA), to define the proportion of Th17 cells, the cells were activated by incubating with phorbol 12-myristate (PMA; 200 ng/mL, P6741, Solarbio, Beijing, China) and ionomycin (1 *μ*g/mL, I8800, Solarbio, Beijing, China) for 6 hours. After 30 min of incubation, brefeldin A (0.7 *μ*L/mL, 00-4506-51, eBioscience, CA, USA) was added. After exclusion of doublets, Molecular Probes (Invitrogen, OR, USA) staining was employed to exclude dead cells. Finally, cells were marked with APC-anti-CD3 (17-0032-82, eBioscience, CA, USA) and PE-anti-CD8 mAb.

### 2.7. Immunofluorescence Staining

The specific procedures for immunofluorescence staining and the drugs involved in the experiment were as reported by our laboratory previously [38].

### 2.8. Serum Cytokine Production

After suffering reperfusion for 6 hours, the peripheral blood and kidneys were isolated for cytokine measurement. The contents of cytokines (IL-10 and IL-17) in serum and kidney tissues were determined using enzyme-linked immunosorbent assay (ELISA) kits according to the manufacturer's instructions (rat IL-10 ELISA kit, E-EL-R0016c; rat IL-17 ELISA kit, E-EL-R0566c, Elabscience Biotechnology Co., Ltd., Wuhan, China). The reactions were measured at 450 nm using a microplate reader (Dynatech, NY, USA), and data are presented as pg/ml.

### 2.9. Western Blotting

Cx43 protein expression in peripheral blood mononuclear cells (PBMCs) was detected by Western blotting [23]. PBMCs were prepared in strict accordance with the method of lymphocyte separation. After that, the samples were lysed using cell lysis buffer (78510, Pierce Biotechnology, Rockford, IL, USA) and crushed by an ultrasonic disintegrator on ice. Then, the supernatant was collected, and a BCA Protein Assay Kit (GK5021, Generay Biotechnology Co., Ltd., Shanghai, China) was used to estimate related protein concentrations. After the samples were pooled and homogenized, equal quality of protein (7 *μ*g) was separated on an SDS-PAGE gel. Proteins were transferred onto a PVDF membrane (Millipore, MA, USA). Membranes were probed with an anti-Cx43 antibody (1 : 1000, ab79010, Cambridge, UK) followed by goat anti-mouse IgG coupled with horseradish peroxidase (1 : 10000, ab6789, Abcam, Cambridge, UK). Next, an ECL kit (Thermo Fisher Scientific, Waltham, MA, USA) was used to detect chemiluminescent according to the manufacturer's instructions, and the membranes were exposed briefly to X-ray film. Protein expression was analyzed using an imaging software program (Gel-Pro Analyzer, Media Cybernetics, USA). The experiment was repeated for three times.

### 2.10. Statistical Analysis

Data are presented as mean ± SEM according to SPSS 17.0 (SPSS Corp, USA). For parametric data, comparisons of different groups were performed by one-way analysis of variance (ANOVA), followed by a post hoc test for multiple comparisons. The Pearson or Spearman product-moment correlation coefficient (*r*) was used to estimate the relationship between the renal injury index and cytokine (IL-17/IL-10) serum and histological levels. Western blot data were analyzed by one-way ANOVA with *t*-test, and the *t*-test was used for statistical comparisons. *p* < 0.05 was considered statistically significant.

## 3. Results

### 3.1. Effects of Estrogen on Renal Function after Renal IRI in Rats

The function of kidney from rats that suffered ischemic injury for 45 min was evaluated by following BUN and Scr levels after renal IRI. Renal tissue showed comparable levels of BUN and Scr between the control and OVX group. After reperfusion for 6 hours, the levels of BUN and Scr in the I/R group were increased dramatically when compared to the control group (*p* < 0.01). Similarly, BUN and Scr levels were significantly higher in the OVX + I/R rats compared with the OVX rats (*p* < 0.01). Moreover, relative to the I/R rats, the concentrations of BUN and Scr were markedly elevated in the OVX + I/R rats (*p* < 0.01). However, this increase was markedly ameliorated by the administration of estradiol (*p* < 0.01) or Gap27 (*p* < 0.05) (Table 1).

### 3.2. Effects of Estrogen on Renal Histopathology after Renal I/R Injury in Rats

**Figure 1:**
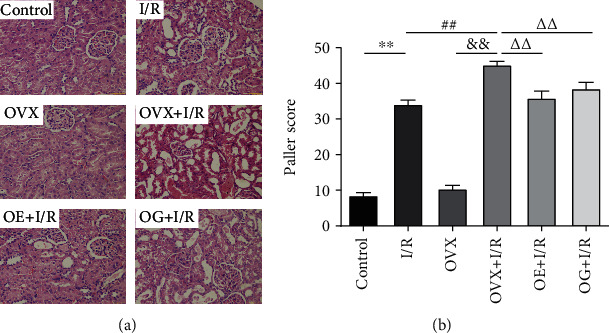
Renal histopathological changes of rats in each group. (a) H&E staining of kidney sections, scale bar: 100 *μ*m. (b) The graph presents quantitative data of renal tissues using Paller scores. Statistical analysis was performed by using one-way ANOVA followed by Tukey's test. Data were presented as mean ± SEM; *n* = 8 per group. ^∗∗^*p* < 0.01 versus control group; ^##^*p* < 0.01 versus I/R group; ^&&^*p* < 0.01 versus OVX group; ^∆∆^*p* < 0.01 versus OVX + I/R group.

Histopathological examinations of renal sections were performed using H&E staining. As shown in Figure 1(a), no obvious morphological changes in renal histology were observed between the control and OVX groups. In the I/R group, proximal tubule expansion, distal tubule without expansion, partial epithelial cell edema, loss of the brush border, and a small amount of inflammatory cells infiltrating in the renal interstitial were observed. These changes were significantly much severer in the OVX + I/R rats, which also exhibited glomerular collapse, proteinaceous cast formation, and extensive tubular epithelial cell necrosis in the cortex and outer medulla. In parallel, estradiol replacement of Gap27 treatment markedly restored these indexes of kidney histological damage. The Paller scoring method was employed to estimate the renal pathological score. The scores in the I/R rats were dramatically increased compared with that of the control rats (34.13 ± 1.20 versus 8.50 ± 0.87, *p* < 0.01). Similarly, Paller scores were significantly higher in the OVX + I/R rats compared to that of the OVX rats (45.18 ± 0.95 versus 10.38 ± 0.98, *p* < 0.01). The score in the OVX + I/R rats was markedly increased relative to the I/R rats (45.18 ± 0.95 versus 34.13 ± 1.98, *p* < 0.01), and these scores were markedly reduced by the estradiol or Gap27 (35.88 ± 1.92 versus 45.18 ± 0.95, *p* < 0.01; 38.56 ± 1.72 versus 45.18 ± 0.95, *p* < 0.01) (Figure 1(b)).

### 3.3. Early Trafficking of CD4^+^ T Lymphocytes into Kidney Tissue after Ischemia Reperfusion

**Figure 2:**
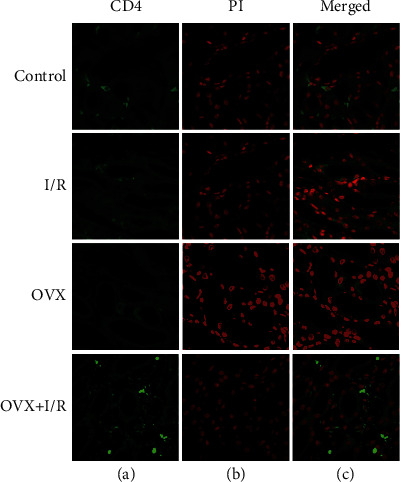
Infiltration of CD4^+^ T cells in kidney tissue. (a) Representative image of positive CD4 expression in kidney tissue. (b) Nuclei are labeled with propidium iodide (PI). (c) Merged images are shown (bar: 10 *μ*m).

To examine T cell infiltration, we performed immunofluorescence staining of renal sections using anti-CD4 antibodies. In control or ovariectomized kidney, there were few CD4^+^ T cells (Figure 2(a), stained green), whereas exactly 6 hours after reperfusion, several CD4^+^ T cells were found in the interstitium (semiquantitative data were not shown here).

### 3.4. Effects of Estrogen on T Cell Subsets in Peripheral Blood of Rats

**Figure 3:**
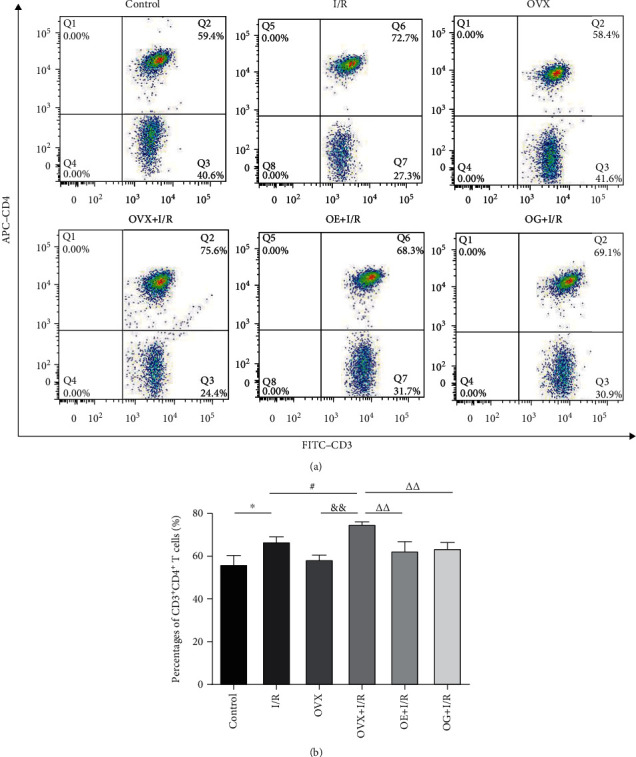
The expression of CD3^+^CD4^+^ T cells in peripheral blood after renal IRI in rats by flow cytometric analysis. (a) Representative flow cytometric scatter plots for CD3^+^CD4^+^ T cell expression. (b) Quantification of the positive rate of CD3^+^CD4^+^ T cells in rats. Statistical analysis was performed by using one-way ANOVA followed by Tukey's test. Data were presented as mean ± SEM; *n* = 6 per group. ^∗^*p* < 0.05 versus control group; ^#^*p* < 0.05 versus I/R group; ^&&^*p* < 0.01 versus OVX group; ^∆∆^*p* < 0.01 versus OVX + I/R group.

**Figure 4:**
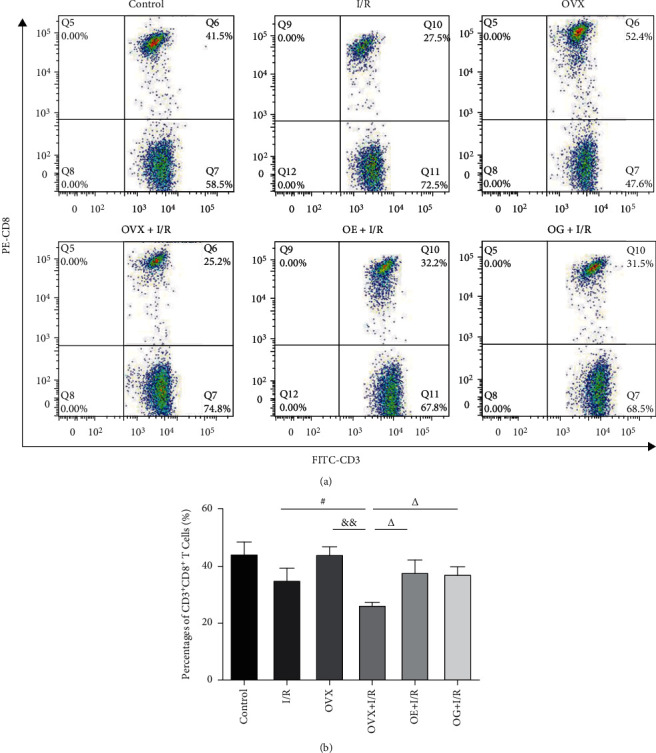
The percentage of CD3^+^CD8^+^ T cells in peripheral blood after renal IRI in rats by flow cytometric analysis. (a) Representative flow cytometric scatter plots for CD3^+^CD8^+^ T cell expression. (b) Quantification of the positive rate of CD3^+^CD8^+^ T cells in rats. Statistical analysis was performed by using one-way ANOVA followed by Tukey's test. Data were presented as mean ± SEM; *n* = 6 per group. ^#^*p* < 0.05 versus I/R group; ^&&^*p* < 0.01 versus OVX group; ^∆^*p* < 0.05 versus OVX + I/R group.

A flow cytometry assay demonstrated that the positive rates of CD3^+^CD4^+^ T cells were dramatically increased in the I/R rats compared to that of the control rats (66.85 ± 2.17% versus 56.23 ± 4.07%, *p* < 0.05). Similarly, CD3^+^CD4^+^ T cells were markedly higher in the OVX + I/R rats than that in the OVX rats (75.04 ± 0.97% versus 58.48 ± 2.00%, *p* < 0.05). CD3^+^CD4^+^ T cells in the OVX + I/R group was dramatically increased compared to that in the I/R group (75.04 ± 0.97% versus 66.85 ± 2.17%), whereas these rates were markedly decreased by administration of estradiol or Gap27 (62.57 ± 4.20% versus 75.04 ± 0.97%, *p* < 0.01; 63.73 ± 2.61% versus 75.04 ± 0.97%, *p* < 0.01) (Figure 3). Nevertheless, as shown in Figure 4, the percentage of CD3^+^CD8^+^ T cells was dramatically decreased in the OVX + I/R rats compared to that in the OVX rats (26.41 ± 0.95% versus 44.28 ± 2.59%, *p* < 0.01), whereas there was no significant difference between the I/R group and control group. The value for the OVX + I/R group was dramatically decreased compared to that of the I/R group (26.41 ± 0.95% versus 35.67 ± 3.72%, *p* < 0.05), but this decrease was markedly restored by the administration of estradiol or Gap27 (37.93 ± 4.30% versus 26.41 ± 0.95%, *p* < 0.05; 37.33 ± 2.53% versus 26.41 ± 0.95%, *p* < 0.05).

**Figure 5:**
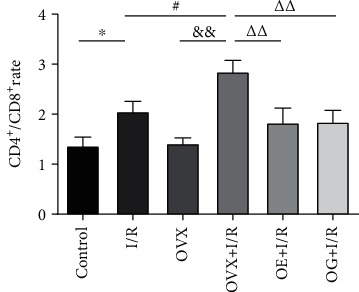
Quantification of the proportion of CD4^+^/CD8^+^ cells in the peripheral blood of rats in each group. Statistical analysis was performed by using one-way ANOVA followed by Tukey's test. Data are presented as mean ± SEM; *n* = 6 per group. ^∗^*p* < 0.05 versus control group; ^#^*p* < 0.05 versus I/R group; ^&&^*p* < 0.01 versus OVX group; ^∆∆^*p* < 0.01 versus OVX + I/R group.

Notably, T cell subset analysis showed an increased rate of CD4^+^/CD8^+^ in the I/R group compared to that of the control group (2.06 ± 0.20 versus 1.37 ± 0.17, *p* < 0.05). A similar trend was observed between the OVX + I/R group and OVX group (2.85 ± 0.22 versus 1.42 ± 0.11, *p* < 0.01). Compared with the I/R group, the rate in the OVX + I/R group was dramatically elevated (2.85 ± 0.22 versus 2.06 ± 0.20, *p* < 0.05), whereas there was a dramatical decrease in the estradiol or Gap27-treated groups (1.83 ± 0.29 versus 2.85 ± 0.22, *p* < 0.01; 1.85 ± 0.23 versus 2.85 ± 0.22, *p* < 0.01) (Figure 5).

### 3.5. Effects of Estrogen on T Cell Subsets in Renal Tissues of Rats

**Figure 6:**
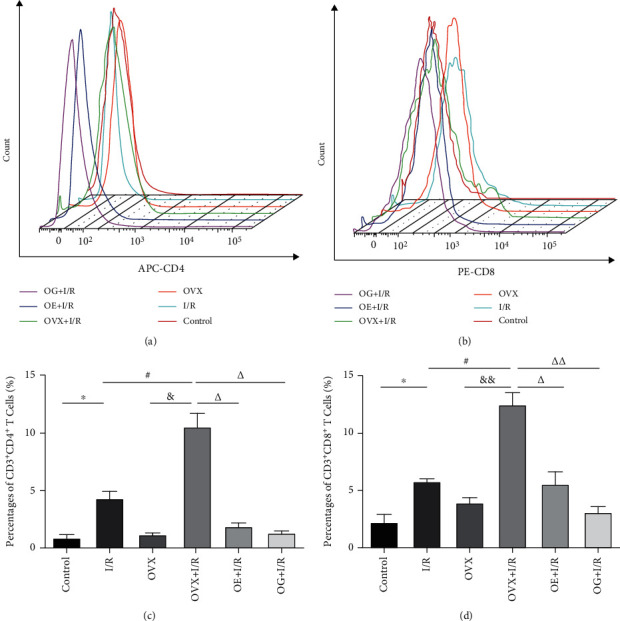
Infiltration of T lymphocyte subsets in the kidney tissue of rats in each group. (a, b) Representative stereoscopic histogram superimposition of CD3^+^CD4^+^ and CD3^+^CD8^+^ T cells in each group. (c, d) Bar graphs of CD3^+^CD4^+^ and CD3^+^CD8^+^ T cell expression ratios in each group of rats. Data are presented as mean ± SEM (*n* = 6). ^∗^*p* < 0.05 versus control group; ^#^*p* < 0.05 versus I/R group; ^&^*p* < 0.05 and ^&&^*p* < 0.01 versus OVX group; ^∆^*p* < 0.05 and ^∆∆^*p* < 0.01 versus OVX + I/R group.

The trafficking of CD4^+^ and CD8^+^ T cell subsets into the kidneys was examined by flow cytometry (Figures 6(a) and 6(b)). The proportions of both CD4^+^ and CD8^+^ T cells were dramatically increased in I/R rats compared to that of control rats (4.33 ± 0.62% versus 0.88 ± 0.28%, *p* < 0.05; 5.80 ± 0.23% versus 2.24 ± 0.67%, *p* < 0.05). Although the proportions of these cells were also greater in OVX + I/R rats than that in the OVX group (10.58 ± 1.17% versus 1.18 ± 0.13%, *p* < 0.05; 12.53 ± 1.04% versus 3.94 ± 0.44%, *p* < 0.01), we observed a clear reduction in the rates of CD4^+^ and CD8^+^ T cell subsets in the I/R group (4.33 ± 0.62% versus 10.58 ± 1.17%, *p* < 0.05; 5.80 ± 0.23% versus 12.53 ± 1.04%, *p* < 0.05). Nevertheless, it is noteworthy that the percentage of CD4^+^ and CD8^+^ T cell subsets was also decreased in both OE + I/R and OG + I/R rats compared to that in the OVX + I/R rats.

### 3.6. Effects of Estrogen on Cytokine IL-17/IL-10

**Figure 7:**
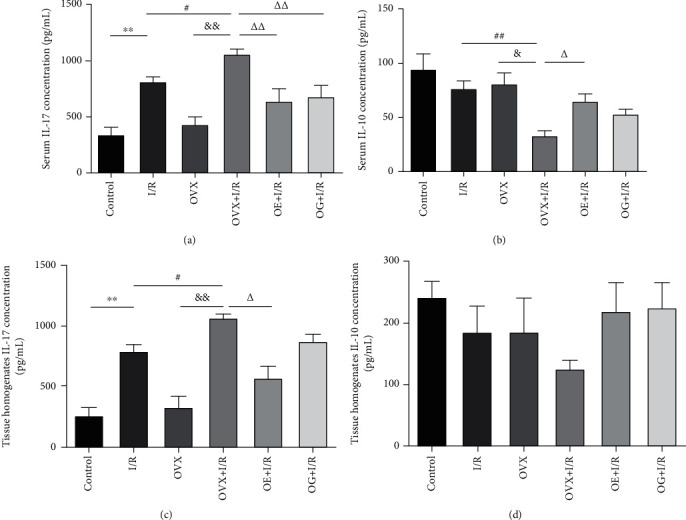
The levels of IL-17 and IL-10 in each group were detected by ELISA. (a) IL-17 was measured in serum collected at 6 hours post reperfusion. (b) IL-10 was measured in serum collected at 6 hours post reperfusion. (c) IL-17 was measured in renal tissue homogenate taken at 6 hours post reperfusion. (d) IL-10 was measured in renal tissue homogenate collected at 6 hours post reperfusion. Data were presented as mean ± SEM (*n* = 6). ^∗∗^*p* < 0.01 versus control group; ^#^*p* < 0.05 and ^##^*p* < 0.01 versus I/R group; ^&^*p* < 0.05 and ^&&^*p* < 0.01 versus OVX group; ^∆^*p* < 0.05 and ^∆∆^*p* < 0.01 versus OVX + I/R group.

To investigate the activation of CD4^+^ T cell subpopulations after IRI, the cytokines IL-17 and IL-10 were examined by ELISA. As illustrated in Figures 7(a) and 7(c), compared with rats suffering IRI, levels of IL-17 in both serum and tissue homogenate were increased significantly in ovariectomized rats subjected to I/R but were decreased significantly in rats treated with estrogen or gap 27. Conversely, serum IL-10 was decreased in ovariectomized rats subjected to I/R compared with rats after IRI, while pretreatment with estrogen or Gap27 increased serum IL-10 as shown in Figure 7(b). Similar trends were observed for IL-10 in tissue homogenate, as shown in Figure 7(d).

### 3.7. Correlation Analysis between IL-17/IL-10 and Kidney Injury Indicators

**Figure 8:**
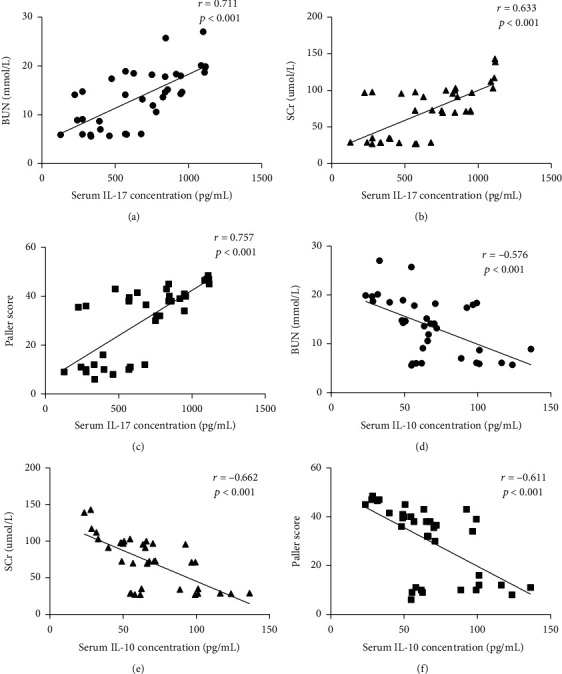
Correlation analysis of serum IL-17 and IL-10 levels with BUN, Scr, and Paller scores. The strength of the correlation was estimated by employing the Pearson product-moment correlation coefficient (*r*) test. The serum concentration of IL-17 was correlated positively with BUN (a), Scr (b), and Paller score (c), whereas the concentration of IL-10 was correlated negatively with BUN (d), Scr (e), and Paller score (f). Each symbol represents a single individual (*n* = 6).

As illustrated in Figures 8(a)–8(c), serum IL-17 levels were positively correlated with BUN (*r* = 0.711, *p* < 0.001), Scr (*r* = 0.633, *p* < 0.001), and renal tubular Paller scores (*r* = 0.757, *p* < 0.001), whereas levels of serum IL-10 were negatively correlated with these indicators, as shown in Figures 8(d)–8(f).

**Figure 9:**
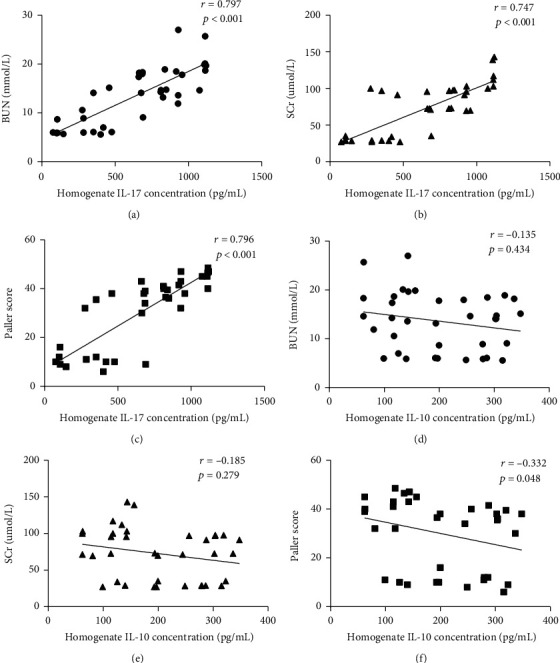
Correlation analysis of homogenate IL-17 and IL-10 levels with BUN, Scr, and Paller scores. The strength of the correlation was estimated by employing the Pearson product-moment correlation coefficient (*r*) test. The homogenate concentration of IL-17 was correlated positively with BUN (a), Scr (b), and Paller score (c), whereas IL-10 was correlated negatively with BUN (d), Scr (e), and Paller score (f). Each symbol represents a single individual (*n* = 6).

Similarly, as shown in Figures 9(a)–9(c), IL-17 in tissue homogenate was positively associated with BUN (*r* = 0.797, *p* < 0.001), Scr (*r* = 0.747, *p* < 0.001), and renal tubular Paller scores (*r* = 0.796, *p* < 0.001), whereas the correlation with IL-10 was insignificant, as shown in Figures 9(d)–9(f).

### 3.8. Estrogen Effect on Cx43 in Peripheral Blood Mononuclear Cells after Renal IRI

**Figure 10:**
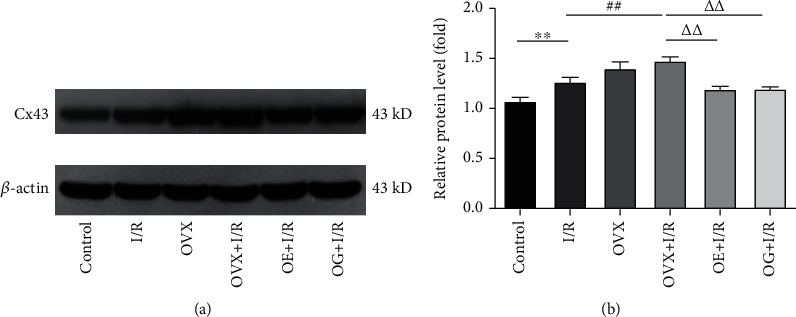
Estrogen inhibits Cx43 expression in peripheral blood mononuclear cells after renal IRI. (a) Western blot analysis of expression of Cx43 protein in each group. (b) Densitometric analysis of Cx43 expression in rats. The data were presented after normalization to *β*-actin and shown as mean ± SEM. ^∗∗^*p* < 0.01 versus control group; ^##^*p* < 0.01 versus I/R group; ^∆∆^*p* < 0.01 versus OVX + I/R group.

Western blot was employed to evaluate the levels of Cx43 in PBMCs (Figure 10(a)). The quantitative analysis suggested that Cx43 protein expression increased significantly in the I/R rats compared to that in the control rats (1.27 ± 0.46 versus 1.07 ± 0.37, *p* < 0.01) (Figure 10(b)). There was an insignificant difference in Cx43 levels in PBMCs between the OVX and OVX + I/R rats. Cx43 levels were markedly higher in the OVX + I/R group compared to that of the I/R rats (1.48 ± 0.38 versus 1.27 ± 0.46, *p* < 0.01). Administration of estrogen or Gap27 alone decreased the expression of Cx43 compared to that in the OVX + I/R rats (1.48 ± 0.38 versus 1.20 ± 0.20, *p* < 0.01; 1.48 ± 0.38 versus 1.19 ± 0.27, *p* < 0.01).

## 4. Discussion

Renal IRI is a complex pathological process that includes primary injury during the ischemic stage and subsequent secondary injury after reperfusion [39]. There is some evidence of a protective effect of female estrogen in several organs subjected to IRI, suggesting that estrogen replacement therapy is an emerging method of preventing and treating IRI [33, 36, 39, 40]. In this study, we clarified that the levels of BUN and Scr (Table 1) and Paller scores (Figure 1(b)) increased significantly after renal IRI. Estrogen depletion in female rats aggravated the above indicators compared with female rats with IRI. Estrogen replacement in the ovariectomized female rats recovered the attenuated I/R-elicited dysfunction and tissue injury of kidney compared with the female rats in an estrogen-depleted state. Taken together, our research shows that estrogen, whether endogenous or exogenous, protects against kidney I/R, which is consistent with the previous studies [33, 39, 40].

There is accumulating evidence indicating that T cells, particularly CD4^+^ T cells, play a critical role in the pathogenesis of renal I/R [10, 12, 13]. However, the mechanism of T cell infiltration during the early phase of renal IRI remains unknown. In an established rat model of kidney IRI, we identified early trafficking of CD4^+^ T lymphocytes to the kidney by immunofluorescence after 6 h of reperfusion (Figure 2). To examine which subtype of T cells is involved in the development of I/R-induced inflammation, we detected the immunologic characteristics of T cells affected by IRI. Our findings demonstrated that CD3^+^CD4^+^ T cells (Figure 3) and the proportion of CD4^+^/CD8^+^ T lymphocytes (Figure 5) in peripheral blood were higher in rats suffering IRI than that in female control rats, whereas CD3^+^CD8^+^ T cells (Figure 4) were lower. Furthermore, we also observed that CD3^+^CD4^+^ T cells (Figure 3) and the proportion of CD4^+^/CD8^+^ T lymphocytes (Figure 5) in peripheral blood were significantly increased in the OVX + I/R rats compared to that in the I/R rats, whereas the rates of CD3^+^CD8^+^ T cells (Figure 4) were markedly decreased. Similarly, both CD4^+^ and CD8^+^ T cells in the kidney were significantly increased in rats suffering IRI, especially in ovariectomized rats (Figure 6). In addition, all of the above changes were reversed upon estrogen replacement or Gap27 intervention. Consequently, these findings suggest that an imbalance of CD4^+^/CD8^+^ T cells, particularly CD4^+^ T cells, is a critical mediator of renal IRI and that estrogen, whether endogenous or exogenous, protects against the renal I/R-mediated inflammation response. Interestingly, our data exhibited that the proportion of CD8^+^ T cells in rats suffering IRI was reduced in the peripheral blood but increased in the kidneys, which may attribute to the decrease in the number of circulating CD8^+^ T cells in the peripheral blood and enhanced infiltration into kidney tissues [36].

Activated CD4^+^ T cells stimulated by various cytokines could usually develop into distinct T helper cell subsets, including Th1, Th2, Th17, and Treg cells. Th17 cells, an important component of adaptive immunity, could secrete IL-22, IL-17A, and IL-17F, which can initiate a powerful inflammatory response in a series of tissues [16]. Th17 cells primarily secrete IL-17A, which contributes to various aspects of acute and chronic inflammation [16, 41]. We used intracellular cytokine staining of IL-17A to define the proportion of Th17 cells. Treg cells play their role in inhibiting immune inflammatory response mainly through direct contact between cells and secretion of some cytokines such as IL-10 and IL-4 [42]. Tregs, major IL-10 producers, exist in various forms and subsets. For specific Treg types, IL-10 production is crucial for their suppressive function. We used intracellular cytokine staining of IL-10 to define the proportion of Treg cells [43]. New insights on the immunopathogenesis of kidney diseases have indicated a role of change of the Th17/Treg axis [18, 44]. Significantly, alterations in the relative abundance of Th17 cells and Tregs have been reported to play a critical role in many models of kidney diseases; however, their roles in renal IRI have not been unequivocal. Regarding CD4^+^ T cell activation, we examined the levels of cytokine IL-17 and IL-10 by ELISA (Figure 7). The results showed that after 6 hours of IRI, the serum and histological levels of IL-17 were significantly increased, suggesting increased activation of Th17 cells, while the content of IL-10 was dramatically decreased, suggesting decreased activation of Tregs, especially in ovariectomized rats. Taken together, our data bring to light an important role of imbalance of Th17/Tregs in IRI-induced renal inflammation. In addition, kidney injury-related indicators were positively correlated with IL-17 levels but negatively correlated with IL-10 levels, suggesting that immune balance is synchronized and associated with renal IRI.

Most recently, increasing evidence has suggested that estrogen could inhibit Th17 cells and block the secretion of the cytokine IL-17 to reduce the inflammatory cascade response induced by IR [36, 45]. Furthermore, emerging reports suggest that estrogen promotes the recovery of kidney injury by amplifying the expression of Tregs [46, 47]. Together, these investigations indicated that elements that influence Th17/Tregs balance may play a crucial role in regulating T lymphocyte-mediated immune homeostasis and further altering inflammatory conditions. In this study, estrogen depletion in female rats aggravated IL-17 levels and decreased IL-10 levels compared with female rats with IRI, and estrogen administration reversed these changes. Those findings reveal that the regulation of estrogen on Th17/Tregs could provide a novel tactics to defend the immune system during renal IRI.

CD4^+^ T cell activation was recently shown to be related to upregulated expression of Cx43, which is ubiquitously expressed in immune cells [28, 48]. During the activation of T cells, the expression of GJCs and HCs, mainly Cx43, results in clonal expansion of T cells [22, 28]. Using Western blot analysis (Figure 10), we demonstrated that the total Cx43 expression was upregulated in PBMCs, especially in ovariectomized rats suffering from renal I/R. When administrated with estrogen, Cx43 expression was dramatically decreased, suggesting that the gap junction protein Cx43 in peripheral blood mononuclear cells including T lymphocytes may play a role in the effects of estrogen on renal IRI. More importantly, we applied Gap 27, a specific inhibitor of Cx43-based channels, to clarify the participation of Cx43-based channels in the regulation of T cell proliferation and secretion of proinflammative cytokines in renal IRI. In this work, after Gap27 intervention, renal function and Paller scores were tremendously ameliorated. Moreover, the positive rates of CD3^+^CD4^+^ T cells and the levels of IL-17 were decreased, and the positive rates of CD3^+^CD8^+^ T cells and the levels of IL-10 were increased. Taken together, our data suggest that Cx43 may be involved in the activation process of estrogen-regulated CD3^+^CD4^+^ T cells and its subset.

In summary, the current study illustrates that administration with 17*β*-estradiol abolishes the development of I/R-initiated inflammation via the regulation of Th17/Treg homeostasis after renal I/R. This regulation is also shown to be correlated with the important role of Cx43 in lymphocytes in immune-mediated renal IRI inflammation. Although the results require future validation, the findings suggest potential new therapeutic options for IRI. It also sets the stage for future studies of our experiments, using fluorescence photobleaching recovery assays to examine the relationship between changes in Cx43 function and estrogen-mediated immune responses.

## Figures and Tables

**Table 1:** Blood urea nitrogen and serum creatinine levels in each group.

Group	BUN (mmol/L)	Scr (*μ*mol/L)
Control	6.49 ± 0.43	30.71 ± 1.57
I/R	16.82 ± 0.95^∗∗^	69.84 ± 1.18^∗∗^
OVX	7.92 ± 0.68	32.92 ± 2.20
OVX + I/R	21.34 ± 1.13^##^	117.38 ± 5.54^##^
OE + I/R	14.48 ± 1.01^&&△△^	63.31 ± 4.82^&&△△^
OG + I/R	15.30 ± 0.60^△^	90.33 ± 4.50^△^

Note: data are shown as mean ± SEM; *n* = 8 per group. ^∗^*p* < 0.05 and ^∗∗^*p* < 0.01 versus control group, ^#^*p* < 0.05 and ^##^*p* < 0.01 versus I/R group, ^&^*p* < 0.05 and ^&&^*p* < 0.01 versus OVX group, and ^∆^*p* < 0.05 and ^∆∆^*p* < 0.01 versus OVX + I/R group.

## Data Availability

All data are availability at Baidu Net disk (link: https://pan.baidu.com/s/1iuVZ9gy86rkBo5R4B1zHkA;extraction code: a123).
