# Correction to “Estrogen Protects against Renal Ischemia‐Reperfusion Injury by Regulating Th17/Treg Cell Immune Balance”

**DOI:** 10.1155/dim/9815037

**Published:** 2026-06-30

Y. Zhang, Y. Chang, Z. Han, K. Ma, X. Zeng, and L. Li, “Estrogen Protects against Renal Ischemia‐Reperfusion Injury by Regulating Th17/Treg Cell Immune Balance,” Disease Markers (2022): 1–14, https://doi.org/10.1155/2022/7812099.

There was erroneous duplication within the PI/OVX (b) frame and Merged/OVX (c) frame in Figure 2, Infiltration of CD4+ T cells in kidney tissue. This was inadvertently introduced during the article production process, and the figure should be corrected as follows:

**Figure:**
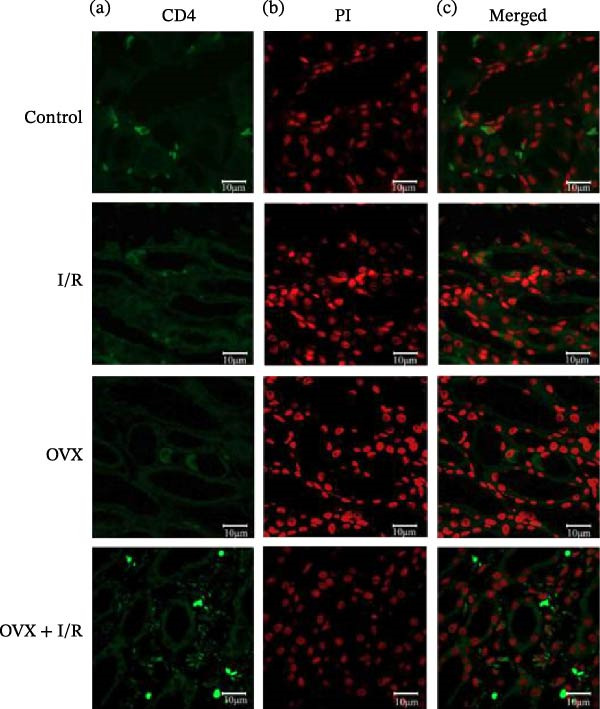


We apologize for this error.

